# Differences in distribution of MLS antibiotics resistance genes in clinical isolates of staphylococci belonging to species: *S. epidermidis, S. hominis, S. haemolyticus, S. simulans* and *S. warneri*

**DOI:** 10.1186/s12866-019-1496-5

**Published:** 2019-06-10

**Authors:** Magdalena Szemraj, Tomasz Czekaj, Jacek Kalisz, Eligia M. Szewczyk

**Affiliations:** 1 Department of Pharmaceutical Microbiology and Microbiological Diagnostic, Medical University of Łódź, Pomorska 137, 90-235 Łódź, Poland; 2 Synevo Sp. z o. o., Microbiological Laboratory of Łódź, Sokola 14, 93-519 Łódź, Poland

**Keywords:** Coagulase-negative staphylococci, *Erm*, MLS_B_, Resistance genes

## Abstract

**Background:**

Macrolides and lincosamides are two leading types of antibiotics commonly used in therapies. The study examines the differences in resistance to these antibiotics and their molecular bases in *S. epidermidis* as well as in rarely isolated species of coagulase-negative staphylococci such as *S. hominis*, S*. haemolyticus*, *S. warneri* and *S. simulans*. The isolates were tested for the presence of the *erm*(A), *erm*(B), *erm*(C), *lnu*(A), *msr*(A), *msr*(B), *mph*(C), *ere*(A) and *ere*(B) genes. Phenotypic resistance to methicillin and mecA presence were also determined.

**Results:**

The MLS_B_ resistance mechanism was phenotypically found in isolates of species included in the study. The most prevalent MLS_B_ resistance mechanism was observed in *S. hominis*, *S. haemolyticus* and *S. epidermidis* isolates mainly of the MLS_B_ resistance constitutive type. Macrolide, lincosamide and streptogramin B resistance genes were rarely detected in isolates individually. The *erm*(B), *ere*(A) and *ere*(B) genes were not found in any of the strains. The *erm*(A) gene was determined only in four strains of *S. epidermidis* and *S. hominis* while *lnu*(A) was seen in eight strains (mainly in *S. hominis*). The *erm*(C) gene was present in most of *S. epidermidis* strains and predominant in *S. hominis* and *S. simulans* isolates. The examined species clearly differed between one another in the repertoire of accumulated genes.

**Conclusions:**

The presence of genes encoding the MLS_B_ resistance among CoNS strains demonstrates these genes’ widespread prevalence and accumulation in opportunistic pathogens that might become gene reservoir for bacteria with superior pathogenic potential.

## Background

*S. epidermidis* are currently the most frequently isolated species among coagulase-negative staphylococci (CoNS) from clinical materials [1–4]. However, also other species of this group such as *S. haemolyticus*, *S. hominis*, *S. warneri* and *S. simulans* have recently been gaining significance [5–9]. They substantially contribute to developing vascular and foreign body-related infections by adhering to tissues and forming biofilm, thus impairing the efficiency of antibiotic treatment. These opportunistic pathogens are primarily isolated from immunocompromised patients as well as from patients undergoing invasive medical treatment. CoNS strains also constitute a therapeutic challenge due to their increasing frequency of the acquisition of the resistance to commonly used drugs [1, 3, 10–15]. *S. haemolyticus* strains have been described as having extensive antibiotic resistance [16]. However, some CoNS species such as *S. simulans* are rarely isolated and generally overlooked during regular diagnosis [7]. Other species like *S. warneri* appear unexpectedly as etiological agents of serious infections [8, 17, 18].

Macrolides and lincosamides occupy one of the leading positions among antibiotics used in therapy, especially in an outpatient treatment. Their role has substantially increased due to the emergence of methicillin-resistant staphylococci and the fact that they also provide an alternative for people allergic to β-lactam antibiotics [19, 20]. As a result, various types of bacteria have acquired resistance to macrolides and this phenomenon has become a global issue. Due to the fact that the binding site of streptogramins B and lincosamides overlaps that of macrolides, the resistance to macrolides often leads to cross-resistance between macrolides, lincosamides and streptogramins B (MLS_B_ resistance phenotype). Many genes limiting the activity of these antibiotics have been described. They affect the expression of several mechanisms of resistance i.e. the methylase production which is responsible for the methylation of the adenine of the 23S rRNA ribosomal subunit as well as active efflux and the production of antibiotic-inactivating enzymes [21, 22]. The phenotypic expression of resistance genes can either be constitutive or inducible and as a result cause additional therapy limitations [21, 23].

As there is no universal mechanism or set of genes determining MLS_B_ resistance, it is difficult to determine the extent to which horizontal gene transfer contributes to the prevalence of this form of resistance when compared with clonal spread. It is known that genes determining MLS_B_ resistance are often incorporated in chromosomal cassettes and might be carried in plasmids what indicates that there is a possibility of their horizontal transfer [24–26].

The mechanism of MLS_B_ resistance as well as the genes responsible for this phenomenon have been thoroughly studied in *S. aureus* and *S. epidermidis*. However, little is known about the prevalence and the genetic bases of MLS_B_ resistance genes in other CoNS species. In order to address this knowledge gap, the aim of this study is therefore to clarify the MLS_B_ resistance mechanisms in rarely isolated CoNS species such as *S*. *haemolyticus, S. hominis, S. warneri* and *S. simulans*.

## Methods

### Bacterial strains

A total of 97 clinical isolates were included in the study. All isolates tested in the study were obtained by a diagnostic microbiology laboratory (Synevo Sp. z o.o.) of Łódź area, Poland since 2014 to 2016. The strains were added to the collection of Pharmaceutical Microbiology and Microbiological Diagnostics Department, Medical University of Łódź and belonged to the following species: *S. epidermidis*, *S. hominis*, *S. haemolyticus*, *S. warneri* and *S. simulans*. However, while all *S. epidermidis* and *S. hominis* strains were obtained from blood samples, the other CoNS species derived from a range of clinical specimens including blood, ear swabs, conjunctival sacs, samples from the genitourinary system, abscesses or skin lesions. Seventeen strains were identified as *S. epidermidis*, 23 as *S. haemolyticus*, 19 as *S. hominis*, 20 as *S. warneri* and 18 as *S. simulans*. The isolates were identified both by means of the MALDI-TOFF technique and genetic methods [27, 28]. The control strains used in PCR identification assays were as follows: *Staphylococcus epidermidis* ATCC 12228, *S. haemolyticus* ATCC 29970, *S. warneri* ATCC 27836, *S. simulans* ATCC 27848 and *S. hominis* subsp. *hominis* ATCC 27844.

### Detection of phenotypic resistance to methicillin and the MLS_B_ resistance phenotype

Resistance to methicillin was determined by means of disc diffusion assays using discs with cefoxitin 30 μg (Oxoid, UK) on Mueller-Hinton agar (Emapol, Poland) according to the rules proposed by the European Centre for Disease Prevention and Control (EUCAST). The diameter of the zone of inhibition was evaluated after an 18-h incubation at 35 °C. Interpretation of the diameters of inhibition zones was as follows - for *S. epidermidis*: sensitive when ≥25 mm, resistant when < 25 mm; for *S. aureus* and coagulase-negative staphylococci other than *S. epidermidis*: sensitive when ≥22 mm, resistant when < 22 mm. *S. aureus* ATCC 29213 was used as negative control. *Staphylococcus aureus* ATCC 43300 was used as positive control.

Resistance to macrolides, lincosamides and streptogramins B was determined by means of disc diffusion assays with erythromycin (15 mg) and clindamycin (2 mg) discs (Oxoid, UK) according to EUCAST. The MLS_B_ resistance mechanisms were detected by D-test, by way of placing above mentioned discs 15–20 mm from each other. After the preparation of bacterial suspensions in line with the 0.5 McFarland standard, the bacteria were inoculated on the surface of Mueller-Hinton agar. Subsequently, the discs were added and the plates were incubated at 35 °C for 18 h. Interpretation of the diameters of inhibition zones was as follows - for erythromycin: sensitive when ≥21 mm, resistant when < 18 mm; for clindamycin: sensitive when ≥22 mm, resistant when < 19 mm.

Both resistance to erythromycin and clindamycin indicated the presence of a constitutive type of MLS_B_ resistance (cMLS_B_). The appearance of a flattening zone around the clindamycin confirmed the presence of inducible resistance (iMLS_B_). Isolates that were resistant to erythromycin and sensitive to clindamycin exhibited an MS phenotype. *S. aureus* ATCC 29213 was used as a control strain.

### Detection of the mecA gene and macrolide, lincosamide and streptogramin B resistance genes

Genomic DNA was isolated from the tested isolates within the study by means of Genomic Micro AX *Staphylococcus* Gravity (A&A Biotechnology, Poland) commercial set according to the manufacturer’s protocol. PCR was conducted with primers synthesized by the Genomed Sequencing Laboratory, Poland, specific for the mecA [29], *erm*(A), *erm*(B), *erm*(C), *lnu*(A), *msr*(A), *msr*(B) [23], *mph*(C) [30]*, ere*(A) and *ere*(B) [31] genes. DNA amplification was performed in the thermal cycler (Biometra, Germany). The reaction products were detected by electrophoresis (70 V, 1.5 h) in 1% (w/v) agarose gels containing Midori Green DNA stain (NIPPON Genetics EUROPE, Germany). A CCD camera (Syngen, Poland) was used in order to obtain results. The sizes of the amplification products were determined by the usage of a commercial molecular size marker DraMix or DNA Marker 2 (A&A BIOTECHNOLOGY, Poland). The presence of tested of genes was determined on the basis of the sizes of the amplification products with specific primer. *S. epidermidis* ATCC51625 harboring the mecA gene and 3 *S. epidermidis* strains: *S. epidermidis* 3718INL harboring *erm*(B) and *mph*(C) genes*, S. epidermidis* 1486IG harboring *erm*(A), *erm*(C) and *mph*(C) genes as well as *S. epidermidis* 1923KIINL harboring the *msr* and *lnu*(A) genes were used as positive controls [32].

### Statistical analysis

Statistical differences were conducted using chi-square (χ2) test. *P* value of < 0.05 was considered as significant.

## Results

Phenotypic resistance to methicillin as well as the presence of the mecA gene in all isolates were determined. The resistance to methicillin were identified in 67% of the isolates. All *S. epidermidis* and *S. hominis* isolates were resistant to methicillin and harbored the mecA gene. Eighteen *S. haemolyticus* isolates were phenotypically resistant to methicillin and harbored the mecA gene whereas two strains had mecA gene without phenotypic resistance. The mecA gene was also determined in 6 *S. warneri* and 5 *S. simulans* isolates, however, phenotypic resistance was confirmed respectively only in four and one strains belonging to abovementioned species.

The MLS_B_ resistance mechanism was found in isolates belonging to all species investigated in the study. However, the prevalence of this resistance was not evenly distributed (Table 1).

**Table 1:** The number of isolates with specific phenotypes of studied CoNS species

No. of isolates	E^R^C^R (^cMLS_B_)	E^R^C^S^ (iMLS_B_)	E^R^C^S^	E^S^C^S^
*S. epidermidis* 17	9	1	5	2
*S. hominis* 19	16	3	0	0
*S. haemolyticus* 23	18	0	5	0
*S. warneri* 20	2	2	8	8
*S. simulans* 18	6	2	0	10

E^R^C^R^ – resistant both to erythromycin and clindamycin, E^R^C^S^ – resistant to erythromycin and susceptible to clindamycin, E^S^C^S^ – susceptible both to erythromycin and clindamycin

Some isolates were sensitive to MLS_B_ antibiotics: 2 *S. epidermidis* strains (both of them resistant to methicillin), 8 *S. warneri* isolates (three resistant to methicillin) and 10 *S. simulans* strains (one was resistant to methicillin). The MLS_B_ resistance mechanism was common in *S. hominis, S. haemolyticus* and *S. epidermidis* isolates – respectively in 100, 78 and 59% of isolates. In most cases it was a constitutive type. The inducible type manifesting a D-shape on Mueller-Hinton agar was observed in several isolates of CoNS included in the study, however, in none of the *S. haemolyticus*. Only in *S. warneri* this type of resistance existed in a greater number of strains than the constitutive type. In some *S. epidermidis, S. haemolyticus* and *S. warneri* isolates the MS_B_ phenotype occurred.

Distribution of genes and their phenotypic manifestation varied between the analyzed species. The sets of genes encoding macrolides, lincosamides and streptogramins B resistance compared with phenotypic MLS_B_ resistance in isolated tests were presented in Table 2.

**Table 2:** Phenotypic manifestation and genes encoding the MLS_B_ resistance as well as the methicillin resistance in tested isolates belonging to the species: *S. epidermidis*, *S. hominis*, *S. haemolyticus*, *S. warneri* and *S. simulans*

Resistance genes	Phenotype (No. of isolates)	No. of isolates/No. of isolates with mecA gene				
		*S. epidermidis*	*S. hominis*	*S. haemolyticus*	*S. warneri*	*S. simulans*
*erm*(A)	cMLS_B_(1)	1/1				
	iMLS_B_(0)					
	MS_B_(0)					
*erm*(C)	cMLS_B_(20)	6/6	8/8		1/1	5/3^a^
	iMLS_B_(3)	1/1			1/1	1/0
	MS_B_(3)	3/3				
*erm*(A),*erm*(C)	cMLS_B_(2)	2/2				
	iMLS_B_(0)					
	MS_B_(0)					
*erm*(C),*msr*(A)	cMLS_B_(2)			1/0		1/1
	iMLS_B_(2)				1/1	1/1^a^
	MS_B_(0)					
*erm*(C),*msr*(A),*msr*(B)	cMLS_B_(1)				1/1	
	iMLS_B_(0)					
	MS_B_(0)					
*erm*(C),*msr*(A),*mph*(C)	cMLS_B_(2)			2/2		
	iMLS_B_(0)					
	MS_B_(0)					
*erm*(C),*mph*(C)	cMLS_B_(1)		1/1			
	iMLS_B_(0)					
	MS_B_(0)					
*erm*(C),*lnu*(A)	cMLS_B_(1)		1/1			
	iMLS_B_(1)		1/1			
	MS_B_(0)					
*erm*(C),*msr*(A),*lnu*(A)	cMLS_B_(1)		1/1			
	iMLS_B_(0)					
	MS_B_(0)					
*erm*(C),*msr*(A),*msr*(B),*lnu*(A)	cMLS_B_(1)		1/1			
	iMLS_B_(0)					
	MS_B_(0)					
*erm*(C),*msr*(A),*mph*(C),*lnu*(A)	cMLS_B_(2)		1/1	1/1		
	iMLS_B_(0)					
	MS_B_(0)					
*erm*(C),*msr*(A),*msr*(B),*mph*(C)	kMLS_B_(7)		1/1	6/6		
	iMLS_B_(1)		1/1			
	MS_B_(0)					
*erm*(C),*msr*(B),*mph*(C)	cMLS_B_(1)			1/1		
	iMLS_B_(0)					
	MS_B_(0)					
*erm*(A),*msr*(B),*mph*(C)	cMLS_B_(1)		1/1			
	iMLS_B_(0)					
	MS_B_(0)					
*msr*(A)	cMLS_B_(2)			2/1		
	iMLS_B_(1)				1/0	
	MS_B_(0)					
*msr*(A),*msr*(B)	cMLS_B_(0)					
	iMLS_B_(0)					
	MS_B_(2)				2/0	
*msr*(A),*msr*(B),*mph*(C)	cMLS_B_(3)			3/2		
	iMLS_B_(0)					
	MS_B_(8)	2/2		3/2	3/2^a^	
*msr*(A),*msr*(B),*mph*(C),*lnu*(A)	cMLS_B_(1)					
	iMLS_B_(0)					
	MS_B_(1)			1/1		
*msr*(A),*mph*(C)	cMLS_B_(2)			2/2^a^		
	iMLS_B_(1)		1/1			
	MS_B_(1)				1/0	
*mph*(C),*lnu*(A)	cMLS_B_(1)		1/1			
	iMLS_B_(0)					
	MS_B_(0)					
*mph*(C)	cMLS_B_(0)					
	iMLS_B_(0)					
	MS_B_(2)			1/1^a^	1/0	

^a^isolates harbor the mecA gene without phenotypic resistance (*S. haemolyticus*: one isolate with *msr*(A),*mph*(C) and one isolate with *mph*(C); *S. warneri*: one isolate with *msr*(A),*msr*(B),*mph*(C); *S. simulans*: two isolates with *erm*(c) and one isolates with *erm*(C),*msr*(A))

The e*rm*(C) gene predominated in all isolates with the MLS_B_ resistance mechanism and this was statistically significant in all species (*p* values were as follows: 0.00001 for *S. epidermidis,* 0.01 for *S. hominis,* 0.00001 for *S. haemolyticus,* 0.0001 for *S. warneri* and 0.0002 for *S. simulans*). The e*rm*(C) gene was present solely or together with other resistance genes in 12 out of 15 resistant strains *S. epidermidis*. Similarly to *S. epidermidis*, MLS_B_ resistant *S. hominis* and *S. haemolyticus* isolates also accumulated many genes. The *erm*(A) gene was present in *S. epidermidis* and *S. hominis* isolates, while *lnu*(A) only in *S. hominis* and *S. haemolyticus*. There were no such genes in other species. The MLS_B_ resistance mechanism appeared also in 3 *S. hominis* isolates and 7 *S. haemolyticus* isolates that do not possess the *erm* gene. Among the examined *S. simulans* and *S. warneri* isolates the MLS_B_ resistance mechanism was less frequent and associated with fewer number of genes. However, the *erm*(C) gene was also predominant. Additionally, there were detected: the *msr* gene family in isolates of *S. simulans*, while the *msr* and *mph* gene families in *S. warneri*. In the examined *S. warneri* isolates, the resistance to macrolides and streptogramins B (MS_B_) was frequently associated with the presence of the *mph*(C) and *msr*(B) genes. The existence of *mph*(C) and *msr*(B) genes in isolates with MS_B_ resistance mechanism was statistically significant (*p* values were as follows: 0.006 for *mph*(C) and 0.0007 for *msr*(B)). The sole *erm*(C) gene was found in 24 out of 97 strains (25%). However, the most prevalent gene was *msr*(A) which was present in 37 of all isolates (38%). In *S. warneri* and *S. haemolyticus* it was occasionally identified as the sole gene. The *msr*(A) gene often coexisted with *msr*(B), *mph*(C) and *erm*(C). Such a set of genes was statistically significant for *S. haemolyticus* (*p* value = 0.0001)*.* The prevalence of *mph*(C), was not evenly distributed among species. It was present in 20 *S. haemolyticus* isolates*,* 7 S*. hominis,* 4 *S. warneri* and 2 *S. epidermidis* whereas it was not detected in *S. simulans* species.

In most cases, MLS_B_ resistance genes coexisted with the phenotypic methicillin resistance encoded by the mecA gene. However, no mecA was detected in 8 *S. warneri*, 4 *S. haemolyticus* and 2 *S. simulans* isolates harboring the macrolides, lincosamides and streptogramins B genes.

## Discussion

In spite of different chemical structures, macrolides, lincosamides and streptogramins B exhibit a similar mechanism of action leading to cross-resistance (MLS_B_) between them [21–23]. The study examines molecular bases of phenotypic manifestation of resistance to macrolides and lincosamides among rarely isolated coagulase-negative staphylococci. This form of resistance is becoming increasingly prevalent and is regarded as being determined by various genes. It may result from the presence of the *erm* family genes encoding the methylase which is responsible for the methylation of adenine in the 23S rRNA ribosomal subunit. For instance, it is known that the *erm*(A) gene is prevalent in *S. aureus,* the *erm*(B) gene in β-hemolytic streptococci and *erm*(X) in corynebacteria and propionibacteria. Resistance to macrolides and streptogramins B (MS_B_ phenotype) can also be determined by the presence of genes responsible for active efflux e.g. the *msr*(A)/*msr*(B) genes in *Staphylococcus* spp., the *mef* gene in *Streptococcus* spp. or *Haemophilus influenzae.* The third mechanism of resistance to antibiotics of the MLS_B_ group is the production of enzymes inactivating a specific substance. These enzymes might be encoded by genes such as *mph*(C) and *lnu*(A) in *Staphylococcus* spp. or by *ere*(A) and *ere*(B) in *Escherichia* and other genera of *Enterobacteriaceae* [33–40].

Resistance to MLS_B_ antibiotics in staphylococci has already been the subject of many studies. However, these studies were mostly limited to the most frequently isolated staphylococci i.e. *S. aureus* and *S. epidermidis* [13, 34, 35, 41–43]*.* Recently, also other staphylococcus species such as *S. hominis*, *S. haemolyticus*, *S. warneri* and *S. simulans*, have appeared as etiological factors of serious human infections. Due to their resistance to antibiotics and ability to form biofilms, these species have become an often underestimated clinical issue. However, the progress in diagnostic laboratory techniques has allowed to see CoNS as a set of specific species instead of a homogeneous group.

Our findings indicate that the MLS_B_ cross-resistance mechanism was present in 61% of all isolates whereas the MS_B_ phenotype was observed in a further 19% of isolates. Li et al. (2015) reported that resistance to erythromycin was more frequent in CoNS isolates than in *S. aureus* and concerned more than half of the strains isolated from mastitis cases in cattle [44]. Similar results were obtained in studies on strains derived from humans. Goudarzi et al. (2016) noted that in isolates obtained from the nasal vestibule, the strains belonging to the CoNS group proved to be more resistant to erythromycin than *S. aureus* [41]. The phenotypic expression of MLS_B_ resistance might be inducible and manifest in clinical resistance to lincosamides and streptogramin B induced by 14- and 15- membered macrolides, or constitutive which determines resistance to all MLS_B_ group antibiotics [21, 23]. Analyzed in our study staphylococcal species form two groups. The first one contains *S. epidermidis, S. hominis* and *S. haemolyticus.* They were isolated from blood and relatively common in clinical laboratory. The greatest number of the isolates of these species exhibited constitutive type of the MLS_B_ resistance. Similar conclusions were drawn by other researchers [35, 45–47]. However, some studies such as these conducted by Dizbay et al. (2008), Teodoro et al. (2012), Szczuka et al. (2016) and Li et al. (2015), indicated that the inducible MLS_B_ resistance might also be commonly present in *S. haemolyticus* and *S. hominis* species [44, 48–50]. The iMLS_B_ mechanism has been frequently reported to occur mostly in *S. aureus* [35, 50, 51]*.* The second group of species investigated in our study consists of *S. warneri* and *S. simulans* which are not often isolated from blood and their pathogenecity potential is weaker. Nonetheless, some of these isolates were also resistant and among *S. warneri* strains the the iMLS_B_ resistance mechanisms were more frequent. The isolates included in our study were tested for the presence of genes responsible for encoding macrolides, lincosamides and streptogramins B resistance in staphylococci: *erm*(A), *erm*(B), *erm*(C), *lnu*(A), *msr*(A), *msr*(B) and *mph*(C)*.* Additionally, the examined isolates were tested for the presence of *ere*(A) and *ere*(B) genes that are described as typical for the *Enterobacteriaceae* family but might also appear in staphylococci [52]. The presence of these genes in staphylococci might indicate transfer of genes between various species and genera of bacteria. The studies regarding CoNS emphasize that the MLS_B_ resistance results mostly from methylase activity and is conditioned by the *erm*(C) gene [35, 45, 53–55]. This gene was frequently detected in the present study, particularly in *S. epidermidis*, *S. hominis* and *S. simulans* isolates. This gene also dominated in the examined by El-Mahdy et.al. (2010) *S. epidermidis* and *S. simulans* strains [56]. Gatermann et.al. (2007) found *erm*(C) as prevalent in *S. haemolyticus* [45]. In our findings *erm*(C) was detected as a sole gene in part of *S. hominis* isolates but in other isolates it coexisted with a range of other genes i.e. *lnu*(A), *msr*(A), *msr*(B) and *mph*(C). El-Mahdy et.al. (2010) found the *msr*(A) gene in *S. hominis* strains as dominant whereas the result obtained in the study conducted by Szczuka et.al. (2016) proved to be similar to ours - the *erm*(C) gene predominated [50, 56].

Resistance to macrolides and streptogramins B (MS_B_) caused by the *msr*(A)/*msr*(B) genes is responsible for active efflux of antibiotics out of bacterial cells. The *mrs*(A) gene was indicated as more prevalent in CoNS than in *S. aureus* [35, 41, 57]. In the presented study, *msr*(A) proved to be the most frequently detected gene. It was present mostly in groups with *msr*(B) and *mph*(C) which condition the synthesis of phosphotransferase. Similarly to the result obtained by Gaterman et al. (2007), the *mph*(C) gene was harbored almost exclusively together with other genes [45]. In our study it was determined as a sole gene in two isolates (*S. haemolyticus* and *S. warneri*) resistant to macrolides. Matsuoka et al. (2003) reported that in *S. aureus* the *mph*(C) gene occurs only if the *msr*(A) gene is also present. The sole presence of the *mph*(C) gene results in a low macrolide resistance [58]. The presence of the *lnu* gene was detected in *S. haemolyticus* and *S. hominis* isolates but not in the *S. epidermidis*, *S. warneri* or *S. simulans* isolates. This gene was frequently identified in *S. haemolyticus* strains by Novotna et al. (2005) and in *S. hominis* isolates by Szczuka et al. (2016) [50, 59]. The *lnu* gene is considered to be responsible for lincomycin resistance and might be present in strains susceptible to clindamycin [59, 60]. The presence of the *ere* genes facilitates the resistance to high concentrations of erythromycin by hydrolysis of the lactone rings [31]. The *ere*(A) and *ere*(B) genes were not detected in any of the isolates tested in the study. However, after PCR with primers applied for *ere*(A) we detected the presence of the product of size corresponding to sought gene in seven isolates of *S. epidermidis*. The result seemed to be reliable as in the studies there was not detected any of unspecific products in sensitive stains. What is more, the primers designed by Sutcliffe et al. (1996) have been already applied to detect this gene in staphylococci [31]. In a study made by Schmitz et al. (2000) that concerned 851 *S. aureus* isolates, *ere*(A) was not identified whereas *ere*(B) was present in less than 1% of all isolates [61]. The same author in other article described the presence of the *ere*(A) gene in one strain of *S. aureus* [62]. The lack of the *ere* genes in *S. aureus* was described by Otsuka et al. (2007) [63]. On the other hand, Li et al. detected the presence of the examined gene in six strains of CoNS and 21 strains of *S. aureus* of bovine milk origin [44]. To our knowledge, it would be the first time when the presence of the *ere*(A) gene in CoNS isolated from humans has been reported. Because of this fact, we have sequenced received PCR products in order to check the homology with *ere*(A) gene that was hitherto mainly detected in bacteria from *Enterobacteriaceae* family. Unfortunately, after the comparison of the sequences in the BLAST program available at http://www.ncbi.nlm.nih.gov/BLAST/ it has turned out that in all seven strains of *S. epidemidis* the obtained sequences are identical but with no homology with the sequence of the *ere*(A) gene. Moreover, the received sequences turned out to be homologous in 98% with the genome fragment of *Staphylococcus capitis* CR01. Our studies revealed that primers designed by Sutcliffe et al. should not be used for staphylococci [31].

Our findings show a correlation between the presence of specific genes or sets of genes and the phenotypic type of the MLS_B_ resistance. The predominant type of the MLS_B_ resistance in *S. epidermidis*, *S. hominis* and *S. simulans* was constitutive. This mechanism was encoded by the *erm*(C) gene as a sole gene or as a part of a group of the genes and it was statistically proved. However, *erm*(C) was also detected in isolates with both inducible MLS_B_ and MS_B_ resistance. Gatermann et al. (2007) reported that the presence of the *erm*(C) gene was associated with the inducible MLS_B_ mechanism in *S. hominis* strains whereas mostly with a constitutive type in other species [45]. Similar results were obtained in the study by Szczuka et al. (2016), in which more than half of the 55 *S. hominis* isolates exhibited the iMLS_B_ mechanism which was associated with the presence of the *erm*(C) gene [50]. In our study, the inducible MLS_B_ mechanism was less frequent than constitutive but both were associated with the presence of the *erm*(C) gene.

Methicillin resistance was observed in all *S. epidermidis* and *S. hominis,* the majority of *S. haemolyticus* isolates and also in minority of *S. warneri* and *S. simulans.* Therefore, it is difficult to evaluate the scope of correlation between the resistance to β-lactam antibiotics and macrolides and lincosamides. However, the in-depth analysis of resistant strains indicate that genes encoding resistance to these antibiotics might be transferred in a common chromosomal cassette [64]. In a study of MRSA strains, Teodoro et al. (2012) reported that the *erm*(A) gene was most commonly present in TN554 as a part of SCC*mec* II or III cassettes whereas in methicillin resistant *S. epidermidis, erm*(C) was located mainly on SCC*mec* IV or nontypeable cassettes [49]. A phenomenon of the common occurrence of the MLS_B_ and methicillin resistance was also analyzed by Castro-Alarco’n (2011) [65]. Bouchami et.al. (2011) indicated the frequent occurrence of the *mec*(A), *erm*(C) and *msr(*A) genes in *S. epidermidis*, *S. haemolyticis* and *S. hominis* isolates derived from blood [66]. Towards increasing resistance to methacillin in staphylococci, including CoNS, the range of antibiotics that are available for treatment of infections caused by these bacteria is getting more limited. The alternative can be macrolides, lincosamydes and streptogramins. Additionally, erythromycin and clindamycin that are recommended for the treatment of the infections of people with ß-lactam allergy [20]. Spreading of the resistance also to such antibiotics may considerably complicate the treatment in the future. Moreover, CoNS might become dangerous genetic reservoir of resistance genes for *S. aureus*.

## Conclusion

Macrolide resistance is rapidly increasing. Therefore, it is reasonable to assume that genes encoding this type of resistance can be transferred between species and genera. However, due to the diversity of the genes involved it is too early to link them with specific mechanisms. Progress in medicine and the increasing contribution of opportunistic pathogens to infections in immunocompromised patients has shifted the profile of the bacterial species responsible for such infections. Apart from *S. epidermidis*, other species such as these described in our study have become more and more frequently isolated. As a result of antibiotic selection such strains which are simultaneously natural human skin microbiota might become dangerous genetic reservoir of resistance genes.

## Data Availability

The datasets used and/or analyzed during the current study are available from the corresponding author on reasonable request.

## Abbreviations

CoNS: Coagulase-negative staphylococci
MLS_B_ resistance: Resistance to macrolides, lincosamides and streptogramins B
